# Supplementary material for: Achieving herd immunity against COVID-19 at the country level by the exit strategy of a phased lift of control
Source: Sci Rep. 2021 Feb 24;11:4445. doi: 10.1038/s41598-021-83492-7 (PMC7904921; doi:10.1038/s41598-021-83492-7)
Supplement: Supplementary file 1 — Supplementary Information 1. [file 41598_2021_83492_MOESM1_ESM.pdf]

Achieving herd immunity against COVID-19 at the country level by the exit strategy of a phased lift of control

Sake J. de Vlas, Luc E. Coffeng*
Department of Public Health, Erasmus MC, University Medical Center Rotterdam, Netherlands

*Corresponding author: l.coffeng@erasmusmc.nl

Supplement 1 – Technical model description

Introduction

This document provides a technical description of the geographically stratified SEIR model used to predict the transmission dynamics of Covid-19 in a country or part of a country. The source code for the model in the form of the R package *virsim* is available online[a] under the CC BY-NC-ND 4.0 license.[b] At the end of the document, Table S1.1 provides a complete overview of the parameter values used in the main analysis. The implications of these parameters (e.g. distributions of local population sizes) are visualized throughout the document as part of the technical description.

Model description

We describe the dynamics of Covid-19 transmission using a standard SEIR model for a closed population of size N, ignoring births and deaths. In terms of ordinary differential equations this can be described as:

$$\frac{\delta S}{\delta t} = -\lambda S$$
$$\frac{\delta E}{\delta t} = \lambda S - \rho E$$
$$\frac{\delta I}{\delta t} = \rho E - \gamma I$$
$$\frac{\delta R}{\delta t} = \gamma I$$

$$\lambda = \beta \frac{I}{N}$$

$$N = S + E + I + R$$

Here, λ is the force of infection, β is the average contact rate in the population (including the average probability that transmission occurs during an average contact), ρ is one over the average incubation time, and γ is one over the average duration of infectiousness, assuming exponentially distributed sojourn times. To relax assumptions about exponentially distributed durations and capture various other heterogeneities (more details below), we implemented the SEIR model in an individual-based framework in discrete time (one-day time steps). We assume that the durations of

[a] http://www.gitlab.com/luccoffeng/virsim
[b] https://creativecommons.org/licenses/by-nc-nd/4.0/

compartments E and I each follow a Weibull distribution with mean ν_E and ν_I and shape α_E and α_I (Figure S1.1). Infection events (transitions from S to E) are assumed to follow an exponential distribution, with the probability of an individual being infected on day t defined as:

$$\Pr_{t,S\rightarrow E} = 1 - \exp\left(-\Delta t \cdot \lambda_t\right)$$

Figure S1.1. Weibull distributions for duration of compartments $\boldsymbol{E}$ and $\boldsymbol{I}$. Duration of compartment $\boldsymbol{E}$ was assumed to follow a Weibull distribution with mean 5.5 and shape 20. Duration of compartment $\boldsymbol{I}$ was assumed to follow a Weibull distribution with mean 10 and shape 0.8. The red dashed line indicates the arithmetic mean durations; dashed black lines indicate the symmetric 95%-confidence intervals.

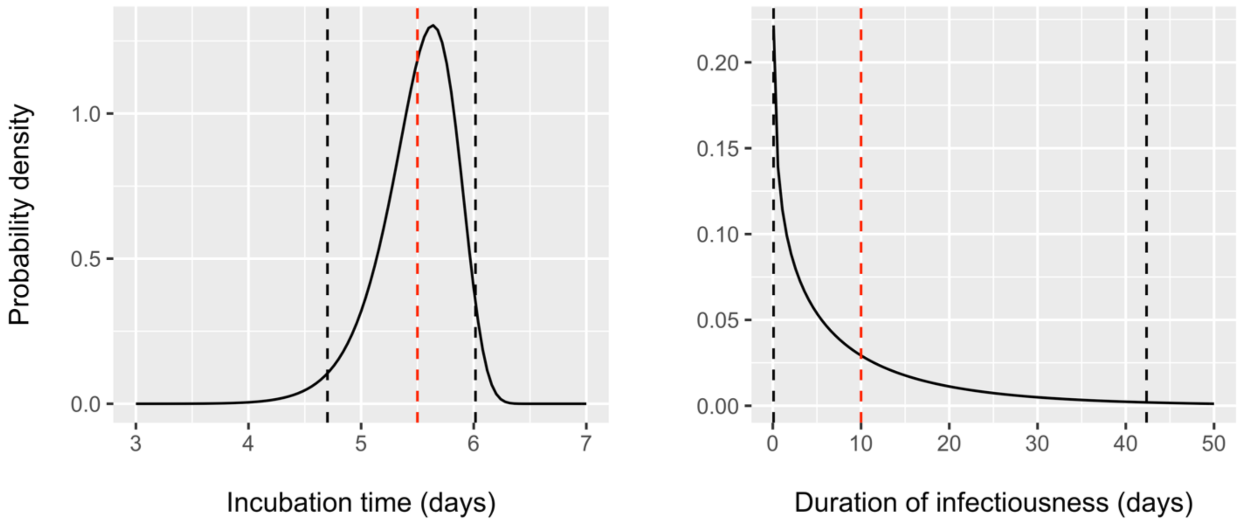

To capture differential mixing of individuals in the same community (e.g. a town, ward or village) and administrative unit (e.g. a province), we distribute all N individuals over K superclusters that each consist of N_k individuals. Within each supercluster, we further subdivide the N_k individuals over J_k clusters of N_{jk} individuals. We allow for variation in cluster size N_{jk} by distributing the population over all $\sum_K J_k$ clusters using a multinomial distribution with cluster-specific probability weights drawn from a log-normal distribution with mean 0 and standard deviation σ (Figure S1.2). We assume that each supercluster contains the same number of clusters (i.e. unrelated to individual cluster sizes), so $J_k = J$, for all $k = 0, \dots, K$.

To capture heterogeneity in contact rates of individuals and potential assortative mixing of individuals with similar transmission-related behavior, we assign each individual a life-long weight w_{ijk} which represents the individual's contact rate relative to the population average contact rate β. We allow relative contact rates to vary between individuals according to a gamma distribution with equal shape and rate α such that average relative contact rate is one (Figure S1.3). Relative contact rates capture inter-individual variation resulting from both contact frequency and the probability of transmission per contact.

Figure S1.2. Variation in cluster population size. The red dashed line indicates the arithmetic mean cluster population size (note that the horizontal axis is logarithmic); dashed black lines indicate the symmetric 95%-confidence interval. The histogram is based on ten thousand simulated cluster sizes with an expected size of one thousand population and $\boldsymbol{\sigma} = \mathbf{0.95}$.

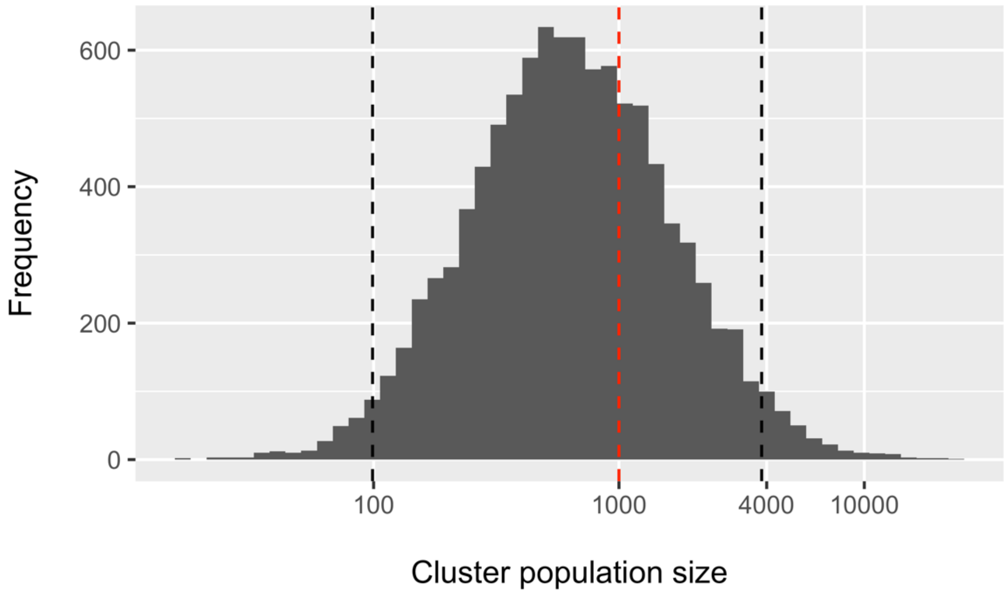

Figure S1.3. Variation in individual relative contact rate. Relative contact rates are assumed to follow a gamma distribution with equal shape and rate ($\boldsymbol{\alpha} = \mathbf{3.4}$) and mean one (red dashed line). Dashed black lines indicate the symmetric 95%-confidence interval.

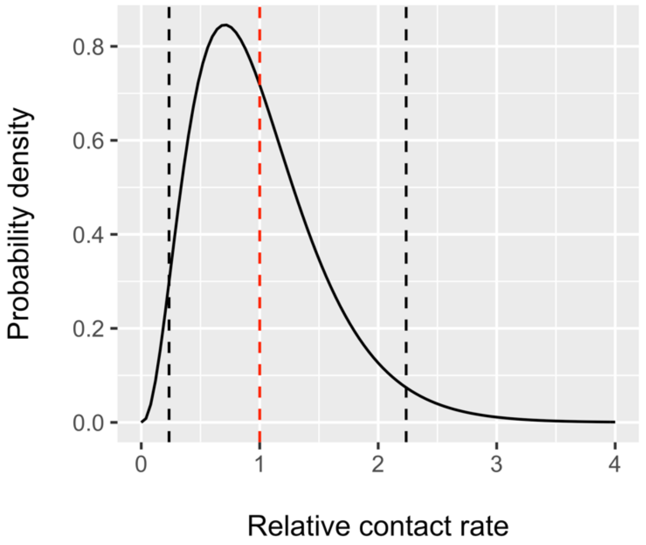

Assortative mixing (i.e. differential mixing of individuals with more similar contact rates) is captured by drawing and assigning individual relative contact rates w_{ijk} as follows. First, after assigning individuals to clusters, for each individual i we draw a random value x_i from the unit normal distribution $N(0,1)$. Likewise, for each cluster j we draw a random value $x_j \sim N(0,1)$. For each individual i in each cluster j we then add up $x_{ij} = x_i + \vartheta \cdot x_j$, where ϑ is a parameter between 0 and 1 representing the level of assortative mixing. We then determine the rank of each individual in the entire population based on their value x_{ij}. Next, we draw N values of $w_{ijk} \sim \Gamma(\alpha, \alpha)$ and order these

(ascending or descending order does not matter). Then finally, we assign each individual the nth of the ordered values of w_{ijk}, where n is the individual's rank in terms of x_{ij} (Figure S1.4). If $\vartheta = 0$, there is no assortative mixing and cluster-level average contact rates are all 1 (± Monte Carlo sampling variation). If $\vartheta = 1$, there is maximum assortative mixing such that individuals' relative contact rates w_{ijk} within a cluster are extremely similar and the cluster-level average contact rates follow a gamma distribution $\Gamma(\alpha, \alpha)$.

Figure S1.4. Variation in the average relative contact rate per cluster due to assortative mixing ($\vartheta = 0.45$). Dashed black lines indicate the symmetric 95%-confidence interval.

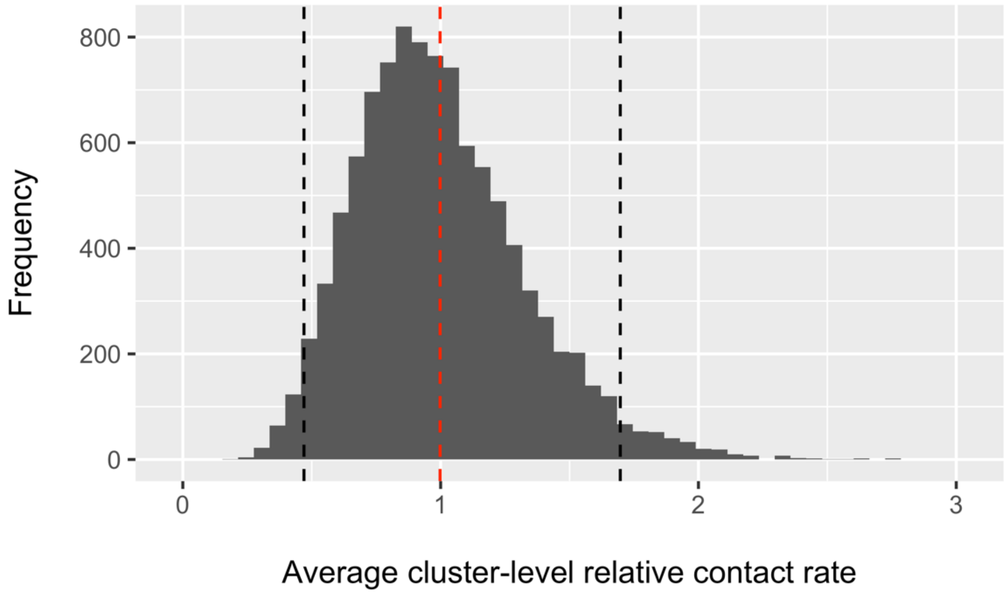

To account for the impact of heterogeneity in individual contact rates, geographical mixing patterns, and potential assortative mixing on transmission, we define the force of infection λ_{ijk} acting on a susceptible individual i in cluster j in supercluster k as:

$$\lambda_{ijk} = \varepsilon_{ik} w_{ijk} \cdot \beta \left((1 - \theta_{\text{SC}} - \theta) \frac{1}{N_{jk}} \sum_{p=1}^{N_{jk}} \Lambda_{pjk} + \theta_{\text{SC}} \frac{1}{N_k} \sum_{q=1}^{J_k} \sum_{p=1}^{N_{qk}} \Lambda_{pqk} + \varphi_k \theta \frac{1}{N} \sum_{r=1}^{K} \varphi_r \sum_{q=1}^{J_r} \sum_{p=1}^{N_{qr}} \Lambda_{pqr} \right)$$

$$\Lambda_{ijk} = \varepsilon_{ik} w_{ijk} \cdot \mathrm{I}(I_{ijk})$$

$$\varepsilon_{ik}^2 \sim \text{Beta}(\mu_k \cdot \tau, [1 - \mu_k] \cdot \tau)$$

Here, $\mathrm{I}(I_{ijk})$ is an indicator function that returns 1 if individual i in cluster j in supercluster k is in the compartment I (i.e. infectious), and 0 otherwise. Interventions aimed at reducing contact rates (e.g. social distancing) are assumed to be implemented at the level of superclusters; their effect ε_{ik} on contact rates may vary between individuals, with its square following a Beta distribution with mean μ and size τ (i.e. the sum of the distribution's shape parameters). In case the reduction of contact rates is the same across and within all superclusters ($\mu_k = \mu$ and $\tau \to \infty$, such that $\varepsilon_{ik} = \sqrt{\mu}$), the quantity $\varepsilon_{ik}^2 = \mu$ represents the reduction in the overall contact rate β (i.e. the quantity reported in the main manuscript). However, to capture the effect of implementing control in only part of the population

(i.e. when $\mu_k \neq \mu$), we define μ_k at the supercluster level. Changes in μ_k over time can be specified per supercluster, allowing the simulation of a geographically heterogeneous intervention. In case μ_k changes over time, individual reductions ε_{ik} in contact rates are assumed to change proportionally to $\sqrt{\mu_k}$, and to be stable over time otherwise (reflecting the individual's inclination to adhere to control). Although not described above, the model also includes a mechanism that allows the user to specify which (random) fraction of the population will take up the intervention at each time point (default value 100%). For individuals who do not take up the intervention, we assume $\varepsilon_{ik} = 1$.

Differential mixing of populations in clusters and superclusters is captured by mixing weights for population-level transmission (θ), supercluster-level transmission (θ_{SC}), and cluster-level transmission ($1 - \theta_{\mathrm{SC}} - \theta$). Isolation of a supercluster for control of transmission is simulated by multiplying a supercluster's contribution and exposure to population-wide transmission by φ (range 0–1).

In absence of variation in individual contact rates (i.e. $w_{ijk} = 1$) and in case of homogeneous mixing of the population ($\theta = 1$, and $\theta_{\mathrm{SC}} = 0$) and uniform implementation of (and adherence to) control measures (i.e. $\varepsilon_{ik} = \sqrt{\mu}$), the above equation for λ_{ijk} can be reduced to the original formulation of the force of infection in a simple SEIR model:

$$\lambda = \mu \cdot \beta \cdot \frac{1}{N} \sum_{k=1}^{K} \sum_{j=1}^{J_k} \sum_{i=1}^{N_{jk}} \mathrm{I}(I_{ijk}) = \mu \cdot \beta \cdot \frac{I}{N}$$

Table S1.1. Overview of parameter values used in the main analysis.

Parameter	Description	Value(s)
ν_E	Average incubation time.	5.5 days
α_E	Shape of Weibull distribution for incubation time.	20, such that the 95%-CI = 4.7–6.0 days
ν_I	Average duration of infectiousness.	10 days
α_I	Shape of Weibull distribution for duration of infectiousness.	0.8, such that the 95%-CI = 0.1–45 days
N	Total population size.	17 million
K	Number of superclusters.	10
J	Number of clusters per supercluster.	1,700
σ	Standard deviation of cluster-level sampling weights for the multinomial distribution of clusters population sizes.	0.95, such that 95% of the clusters are inhabited by 100–4000 individuals, and 0.2% of clusters harbor >10 thousand individuals
β	Overall contact rate.	0.1717, such that the initial exponential growth of the epidemic is equal to one predicted by a homogeneous model with $\beta = 0.25$, $\nu_E = 5.5$, and $\nu_I = 10$ ($R_0 = 2.5$)
α	Shape and rate of gamma distribution for variation in individual relative contact rates (relative to overall contact rate β).	3.4, such that the 2.5th and 97.5th percentiles of the distribution of relative contact rates differ by a factor 10.
ϑ	Level of assortative mixing (range 0–1).	0.45, such that the 2.5th and 97.5th percentiles of the distribution of average relative contact rate in each cluster differ by a factor 3.8.
θ_{SC}, θ	Weights for transmission coming from superclusters and the general population, respectively. The derived weight for transmission coming within cluster is $1 - \theta_{\mathrm{SC}} - \theta$.	0.05, 0.05
μ	The relative level to which transmission is reduced on average in supercluster k.	0.25 for intensive control, 1 otherwise
τ	Size of the Beta distribution for inter-individual variation in the effect of contact-related interventions	∞
φ	Multiplier for the contribution and exposure of a supercluster to population-level transmission in case of isolation.	0.5 in case of isolation of the supercluster, 1 otherwise
