# Supplementary material for: Achieving herd immunity against COVID-19 at the country level by the exit strategy of a phased lift of control
Source: Sci Rep. 2021 Feb 24;11:4445. doi: 10.1038/s41598-021-83492-7 (PMC7904921; doi:10.1038/s41598-021-83492-7)

Achieving herd immunity against COVID-19 at the country level by the exit strategy of a phased lift of control

Sake J. de Vlas, Luc E. Coffeng*
Department of Public Health, Erasmus MC, University Medical Center Rotterdam, Netherlands

*Corresponding author: l.coffeng@erasmusmc.nl

Supplement 2 – Alternative strategies

This document provides an overview of predictions with our model for the course of the COVID-19 epidemic for some alternative strategies: no interventions, an ongoing period of country-wide intensive control, and the “flattening the curve” strategy.

If we let the COVID-19 epidemic run its natural course (Figure S2.1, left panel), approximately 60% of the population will experience infection. Note that this 60% reflects the commonly quoted formula $1-\frac{1}{R_0}=1-\frac{1}{2.5}=60\%$ for the minimum level of herd immunity required to avoid an epidemic. At the peak of a natural epidemic, there will be 60 thousand prevalent cases of infection per million population (6%), which by far surpasses the limits of the health care system. The overall period of surpassing the threshold is about 100 days.

In the middle and right panels of Figure S2.1, we illustrate the impact of intensive control measures for an extended period (one or two years) such that case numbers remain very low and within the limits of the health care system. However, as a result, very little herd immunity is developed as few people experience natural infection. Then, after lifting these intensive control measures (be it after one or after two years), residual circulating virus is likely to still cause an outbreak of similar size to the one that would have occurred without interventions (only one out of the presented eight repeated simulations resulted in elimination). This probability of a delayed outbreak would only increase further if also accounting for reintroductions of the virus from outside the population. This strategy can therefore only be successful if continued until a vaccine is widely available, or if combined with intensive tracking of remaining cases, which may be unfeasible as so many infections pass unrecognized. Flattening the curve may also work when continued until the virus is eliminated from most parts of the world, with strict isolation of countries that are still affected, which we consider unrealistic.

Figure S2.1. Course of the COVID-19 epidemic in case of no interventions or one or two years of country-wide intensive control measures. During intensive control (period indicated by vertical dashed lines) the transmission rate is assumed to be reduced to 25% of its original value. The top row shows the number of prevalent cases of infection based on 8 model runs (thin grey lines) and their average (blue line). The horizontal red dashed line indicates the assumed number of prevalent cases associated with maximum IC capacity. The bottom row shows the distribution of the population over the susceptible (S), exposed (E), infectious/symptomatic (I), and recovered/immune (R) states, based on the average of the same 8 model runs. Simulations were performed for a population of 1 million people distributed across one thousand clusters and ten superclusters**.**

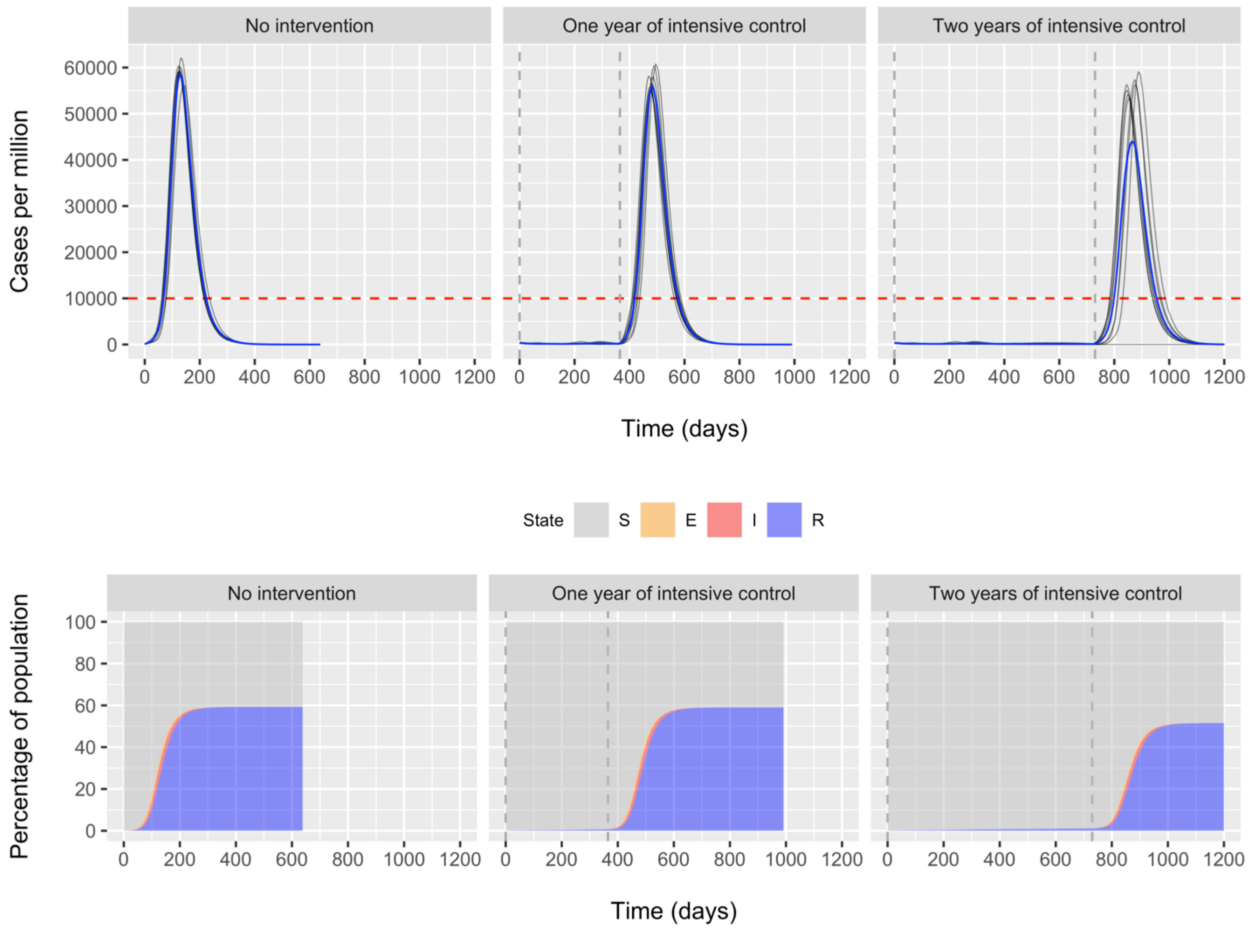

As an alternative, the "flattening the curve" strategy has been proposed to generate herd immunity while keeping the number of prevalent cases within the limits of the health care system (Figure S2.2). However, this strategy comes with three major disadvantages. First of all, the number of prevalent cases is highly sensitive to the actual impact of intervention strategies. If the impact is too high, too little immunity will be developed such that upon lifting of interventions a major epidemic will ensue (left panel of Figure S2.2). If the impact of interventions is too low, the case load will quickly rise above the threshold for what the health care system can manage (right panel). Interventions would have to be carefully titrated such that the maximum case load remains manageable and equal both before and after the intervention (middle panel), which will be challenging. Second, depending on the maximum health care capacity, flattening the curve may take a considerable amount of time (600-800 days for the assumed threshold of 10 thousand prevalent cases of infection) during which the entire population would have to adhere to these carefully titrated control measures. Third, flattening the curve would result in suboptimal use of health care capacity, as at the beginning and end of each bell-shaped epidemic curve, the number of cases is well below the maximum capacity. Ideally, the number of prevalent cases would be constant over time. When flattening the curve, this

would require that (adherence to) control measures is continuously titrated over time (first slowly increasing in intensity, and then slowly decreasing again) to keep the number of cases stable. Clearly, this approach would be extremely difficult to design and to be adhered to by the public.

Figure S2.2. Course of the COVID-19 epidemic during a "flattening the curve" strategy by means of country-wide control measures. During intensive control (period indicated by vertical dashed lines) the transmission rate is assumed to be reduced to the value indicated in the panel header. The top row shows the number of prevalent cases of infection based on 8 model runs (thin grey lines) and their average (blue line). The horizontal red dashed line indicates the assumed number of prevalent cases associated with maximum IC capacity. The bottom row shows the distribution of the population over the susceptible (S), exposed (E), infectious/symptomatic (I), and recovered/immune (R) states, based on the average of the same 8 model runs. Simulations were performed for a population of 1 million people distributed across one thousand clusters and ten superclusters**.**

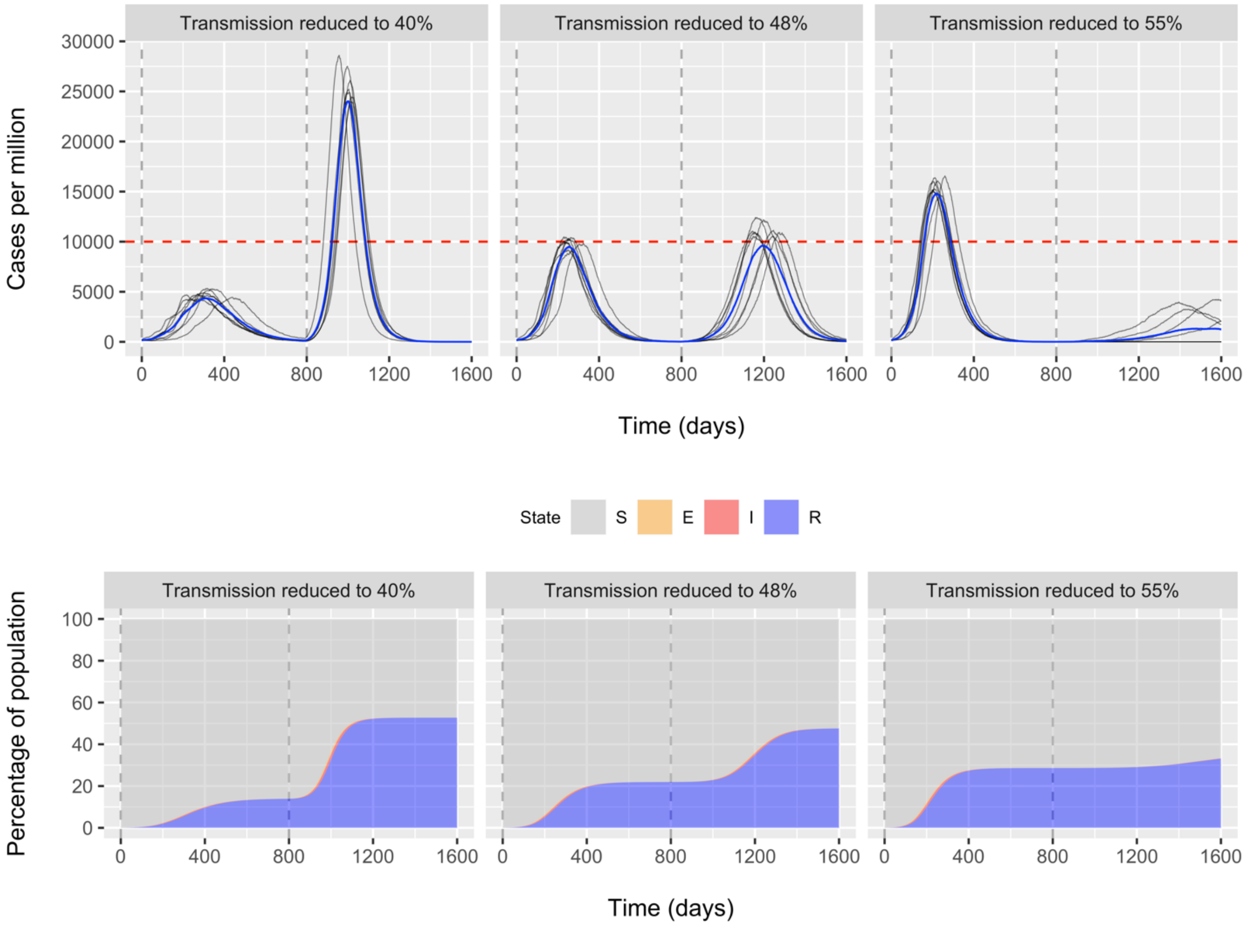

Supplement: Supplementary file 2 — Supplementary Information 2. [file 41598_2021_83492_MOESM2_ESM.pdf]
