# Supplementary material for: Achieving herd immunity against COVID-19 at the country level by the exit strategy of a phased lift of control
Source: Sci Rep. 2021 Feb 24;11:4445. doi: 10.1038/s41598-021-83492-7 (PMC7904921; doi:10.1038/s41598-021-83492-7)
Supplement: Supplementary file 3 — Supplementary Information 3. [file 41598_2021_83492_MOESM3_ESM.pdf]

Achieving herd immunity against COVID-19 at the country level by the exit strategy of a phased lift of control

Sake J. de Vlas, Luc E. Coffeng*
Department of Public Health, Erasmus MC, University Medical Center Rotterdam, Netherlands

Corresponding author: l.coffeng@erasmusmc.nl

Supplement 3 – Strategy adjustments and sensitivity analyses

Introduction

This document provides an overview of model predictions for the course of the COVID-19 epidemic for different scenarios of the proposed strategy of a phased lift of control and sensitivity analyses for the effect of uncertainty in model parameters. All outcomes are compared with the main strategy: a population (e.g. a country) with 10 superclusters (e.g. provinces), containing an equal number of clusters, each with on average 1000 inhabitants. In the first supercluster, the one with the highest initial burden, lifting of control occurs after 15 days, a (random) second supercluster follows 120 days later, followed by a phased lifting of control in another supercluster each 90 days (random order). Each figure panel shows the number of prevalent cases of infection based on 8 model runs (thin grey lines) and their average (blue line). The vertical dashed lines reflect the 10 moments of lifting control. The horizontal red dashed line indicates the assumed number of prevalent cases associated with maximum IC capacity (i.e. 10 thousand per million).

To account for random phenomena, such as the possible occurrence of unwanted local outbreaks, we have increase stochasticity by showing all results in this supplement only for populations of 1 million (thus, 100 clusters per supercluster), instead of the 17 million (i.e. 1700 clusters per supercluster) used in Figure 1 of the main text. Figure S3.1 below illustrates how the random variation is higher in the simulations for a population of 1 million, with two runs slightly exceeding the IC capacity threshold for a short time.

Figure S3.1. Simulations based on a population of 17 million (as Figure 1 in the main text) compared to 1 million.

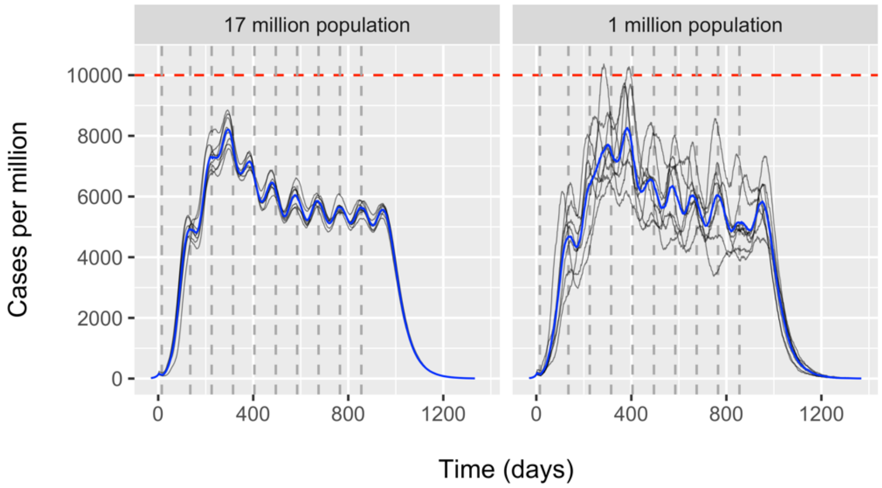

Strategy adjustments

Below we illustrate the different options that decision-makers can consider when implementing phased lifting of control.

Figure S3.2 shows how increasing the number of phases of lifting control (i.e. the number of superclusters) decreases the maximum number of prevalent cases and increases its stability over time. This means that when IC capacity increases sufficiently, control can be lifted in multiple superclusters at once, reducing the overall duration of the strategy.

Figure S3.2. Number of phases.

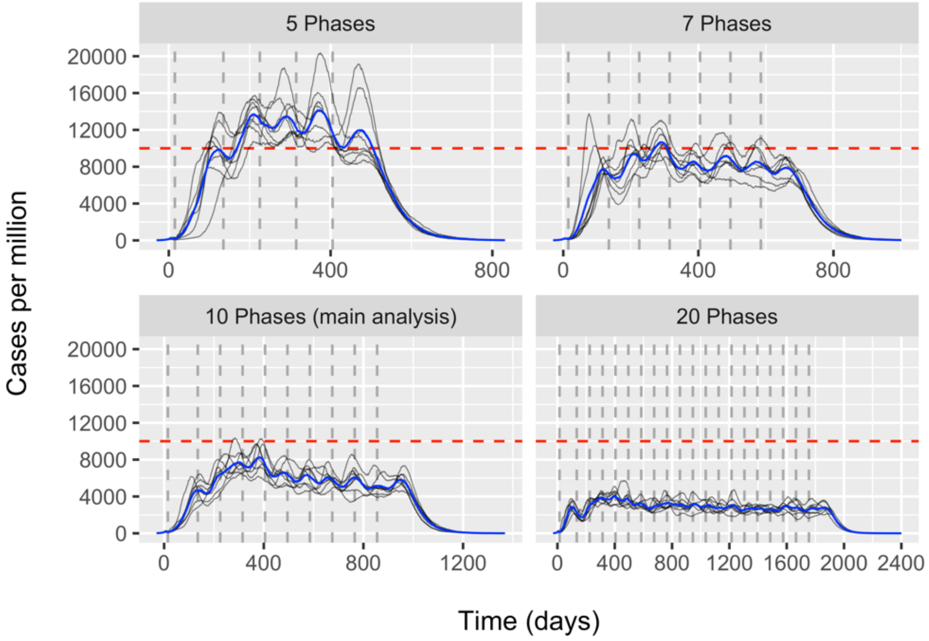

Figure S3.3 shows the minimum condition of sufficient reduction in transmission in superclusters where control has not (yet) been lifted. More intensive control measures (left panel) than assumed in our main analysis (middle panel) do not have much additional impact, but further flatten the curve somewhat. Insufficient control (right panel) would lead to an accumulation of multiple supercluster-level outbreaks at the start of the strategy.

Figure S3.4 shows the benefit of somewhat increasing the first interval (between lift of control in the first and second supercluster) to let the epidemic take off. If this period is shorter (second panel) or too short (left panel), there is a higher risk of multiple supercluster-level outbreaks accumulating during the start of the strategy. Ideally, the second phase is initiated shortly after the peak of the local epidemic in the first supercluster (third panel from the left). Extending the first interval any further than that (right-most panel) leads to an unnecessary overall delay.

Figure S3.5 shows the impact the interval between lifting control in consecutive superclusters from the second phase onwards. Basically, a longer interval stretches the epidemic over time, lowering flattening, and stabilizing the overall course of the epidemic.

Figure S3.3. Effect of control measures in terms of reduction in transmission (when not lifted).

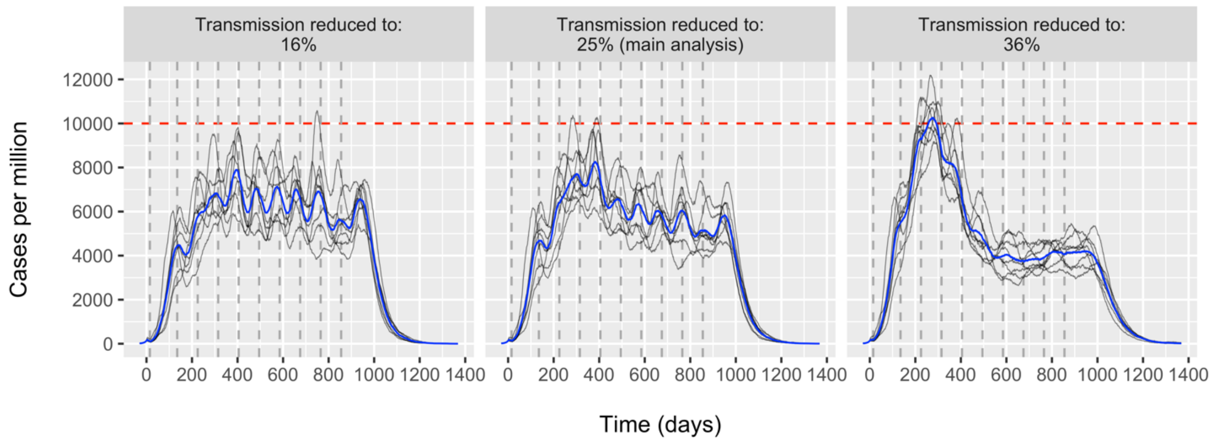

Figure S3.4. Duration of the first interval of phased lifting of control.

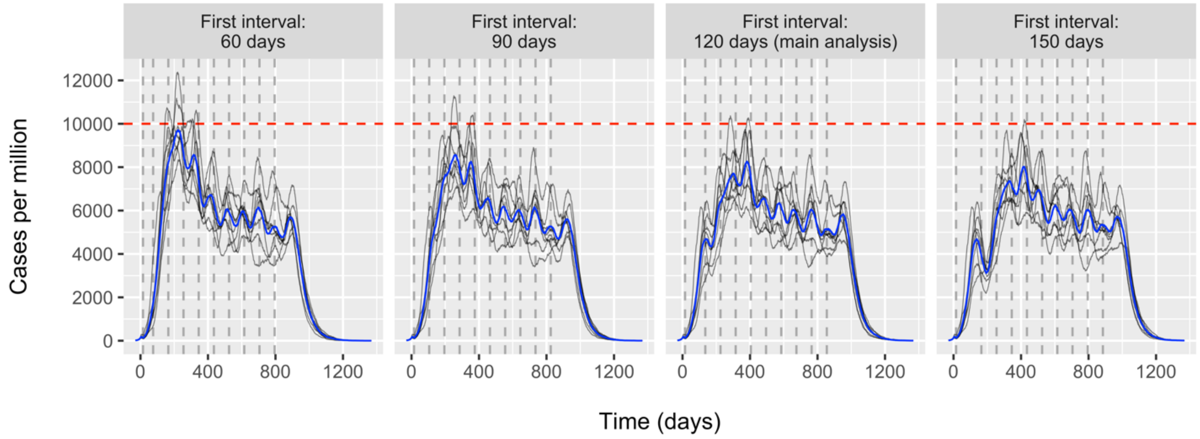

Figure S3.5. Interval between lifting of control in consecutive superclusters from the second onward.

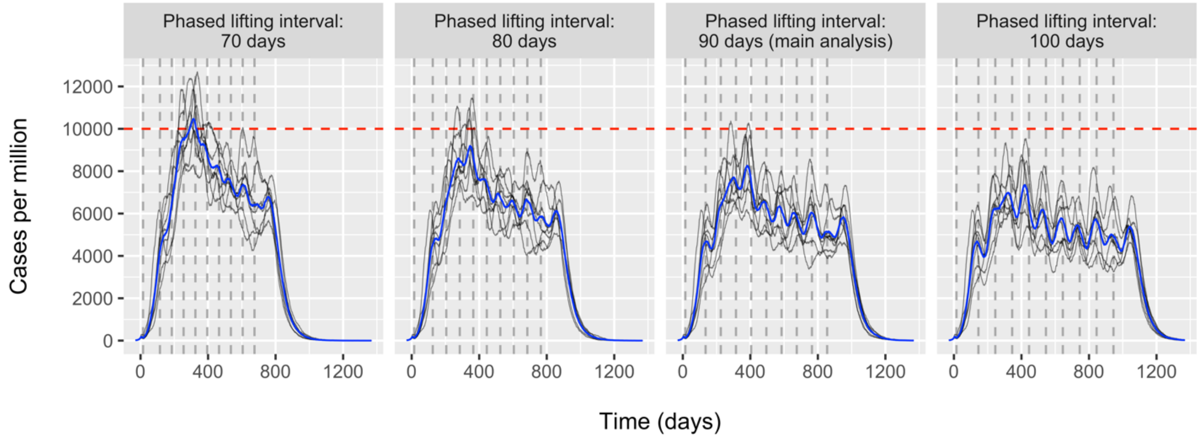

Figure S3.6 shows the importance of starting the phased lift of control in the most affected supercluster (left panel). If control is initially lifted in a random cluster (right panel), there is a high risk of the epidemic in the first supercluster setting off too late, leading to accumulation of several supercluster-level outbreaks. The latter risk could be mitigated by extending the first interval (Figure S3.4), at the cost of a longer duration of the strategy.

Figure S3.6. Choice of supercluster to lift control in first (50 repeated simulation runs).

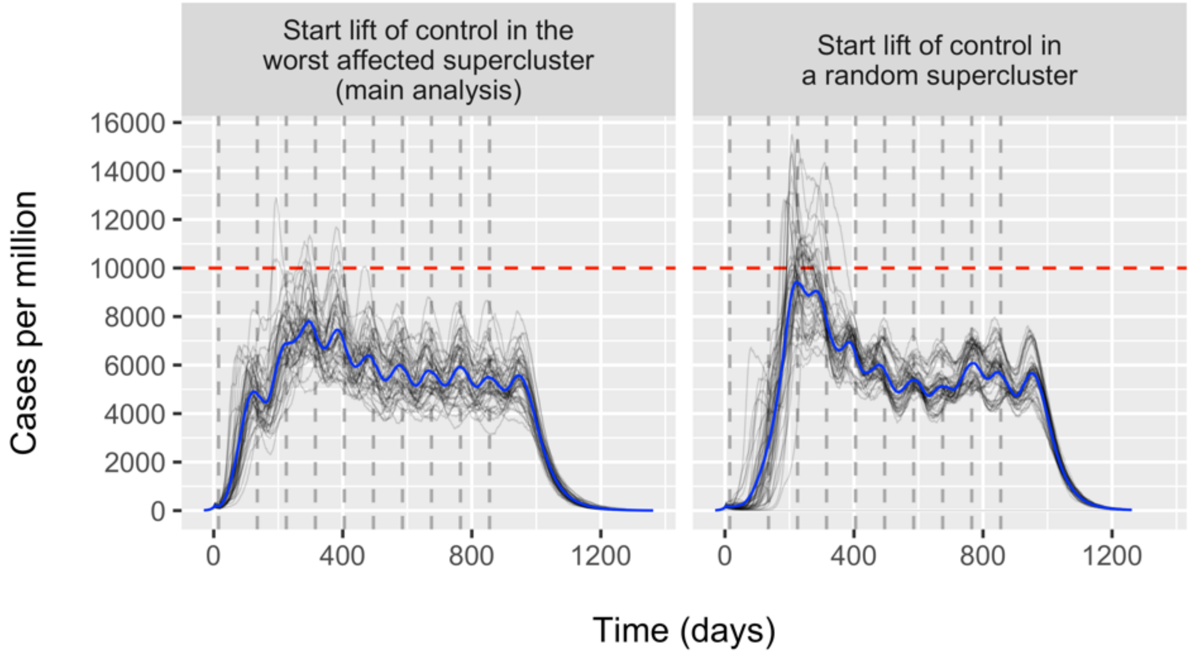

Figure S3.7 demonstrates that isolation of a supercluster while lifting control is not strictly necessary, under the minimum condition of sufficient control measures in superclusters that have not yet lifted control (see also Figure S3.3).

Figure S3.7. Impact of temporary isolation of the supercluster during the phase that control is being lifted.

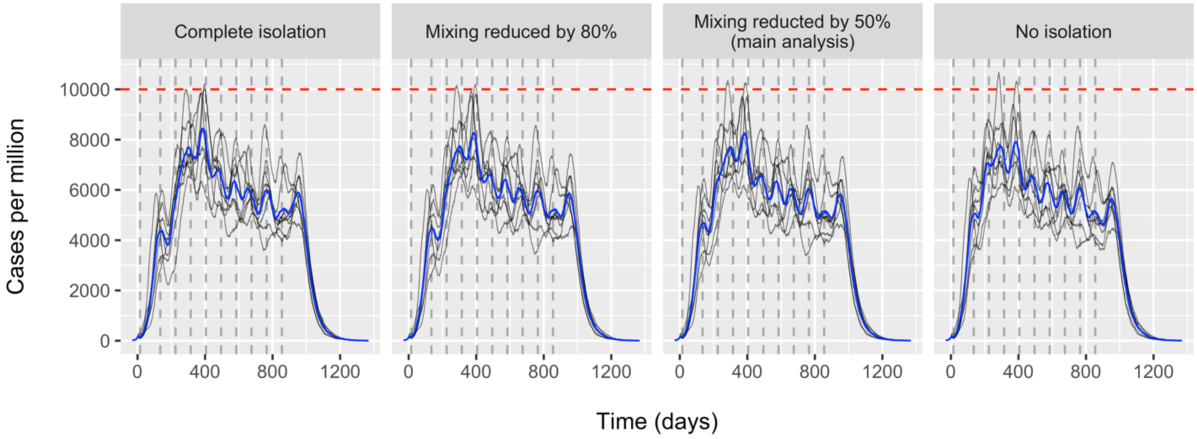

Figure S3.8 demonstrates that it is not strictly necessary that all people in a supercluster return to their normal daily activities and corresponding contact rates. If 25% of the population in a supercluster maintains control measures (e.g. social distancing) during the time interval that control is initially lifted in their supercluster (middle panel), this makes hardly any difference compared with 100% participation as in the main analysis (left panel). However, if 25% of individuals maintains control measures until the end of the strategy, the reservoir of remaining susceptible people may trigger a small epidemic (right panel). Policy should therefore be aimed at mobilizing the population to maximally participate in the phased lifting of control measures, which may be challenging.

Figure S3.8. Population participation in lifting of control measures.

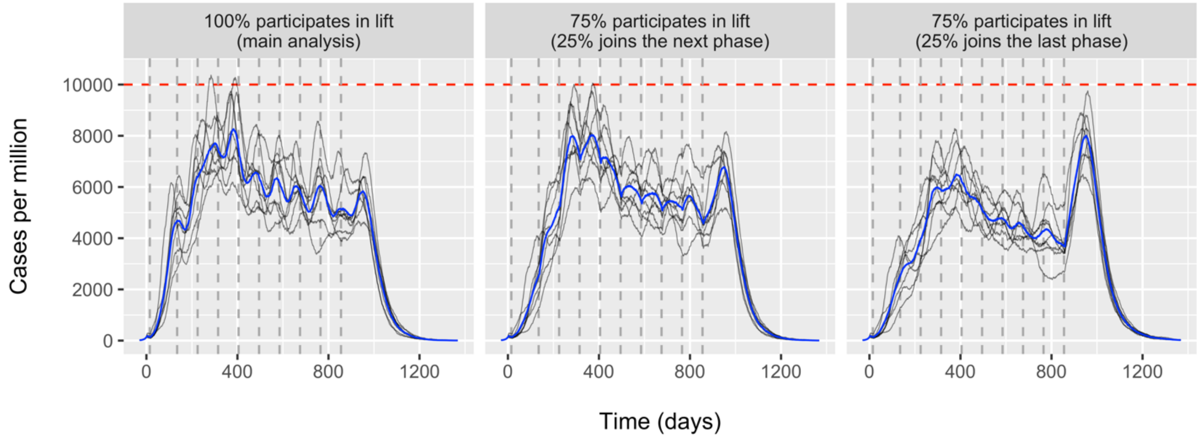

Figure S3.9 shows that phased lifting of control may commence at any moment during the epidemic (provided the minimum conditions are met – see main text). This means that the health care system has time to make the necessary preparations before lifting control in the first supercluster.

Figure S3.9. Duration of initial control measures.

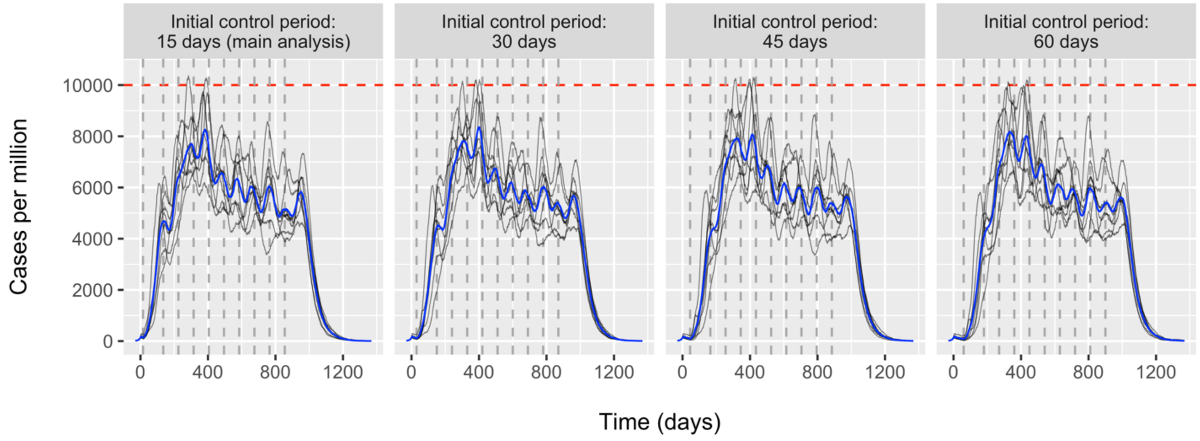

Sensitivity analyses

In this section, we show the impact of varying of all model parameters on the predicted impact of phased lifted of control. We anticipate that more detailed quantification of these parameters will be possible based on data and experience in the near future, which could also be generated by implementing a phased lift of control. We first show results for alternative assumptions regarding the three main parameters of the SEIR model, i.e. the average contact rate and the average durations of exposure (incubation time) and infectiousness.

Clearly, a higher average contact rate (and thus R_0) leads to more cases and higher and more variable epidemic peaks (Figure S3.10), requiring more health care capacity or a strategy with more phases (Figure S3.2). Similar to the average contact rate (Figure S3.10), longer durations of infectiousness lead to more cases (Figure S3.11), requiring higher health care capacity or a strategy with more phases (Figure S3.2). Lower variation in the duration of infectiousness leads to more pronounced peaks in case numbers (Figure S3.12). Uncertainty about the length of and variation in incubation time has little to no effect on the impact of phased lifting of control (Figure S3.13, Figure S3.14). Note that in the left-most panels of Figure S3.10 and Figure S3.12, one out of eight simulations resulted in stochastic fade-out.

Figure S3.10. Impact of R_0 by proportionally changing the average contact rate.

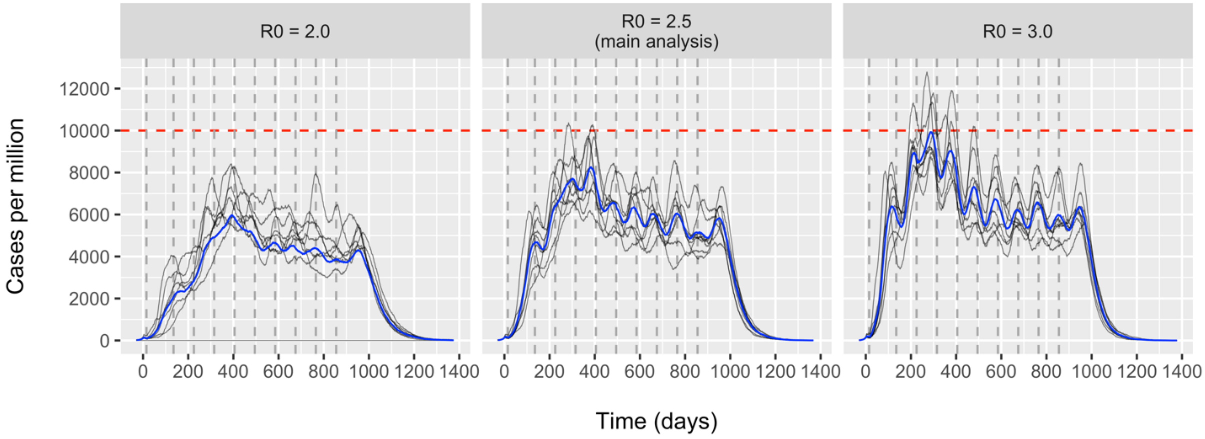

Figure S3.11. Average duration of infectiousness.

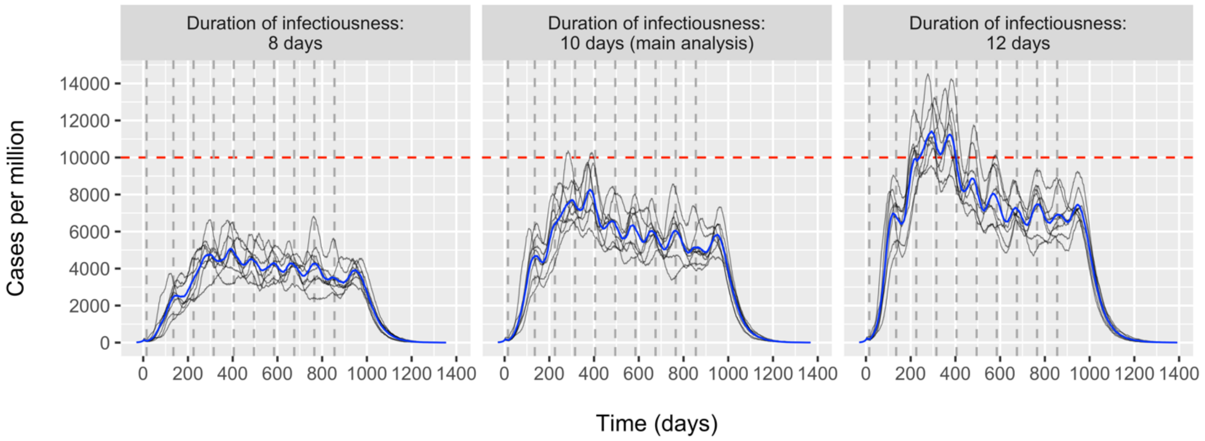

Figure S3.12. Shape of the Weibull distribution for variation in duration of infectiousness. Higher values for shape indicate lower variation.

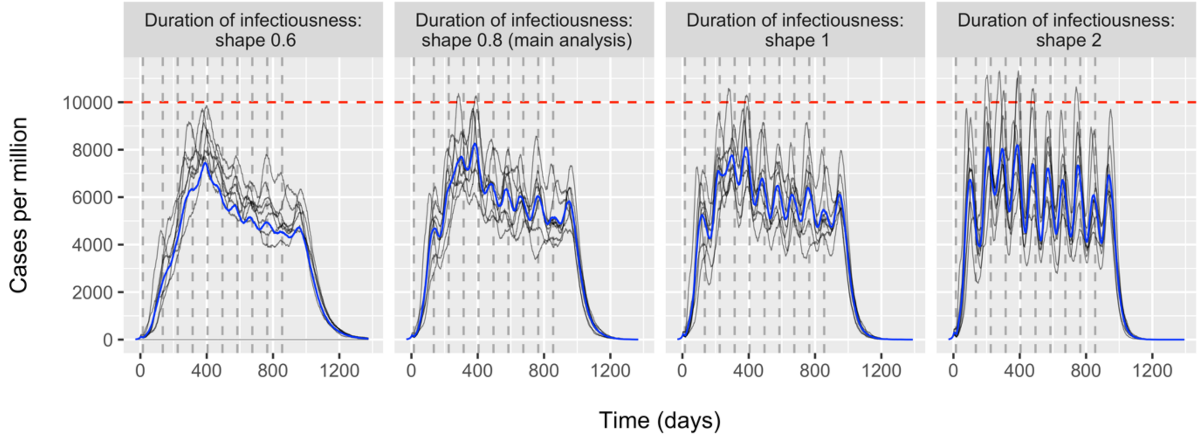

Figure S3.13. Average incubation time.

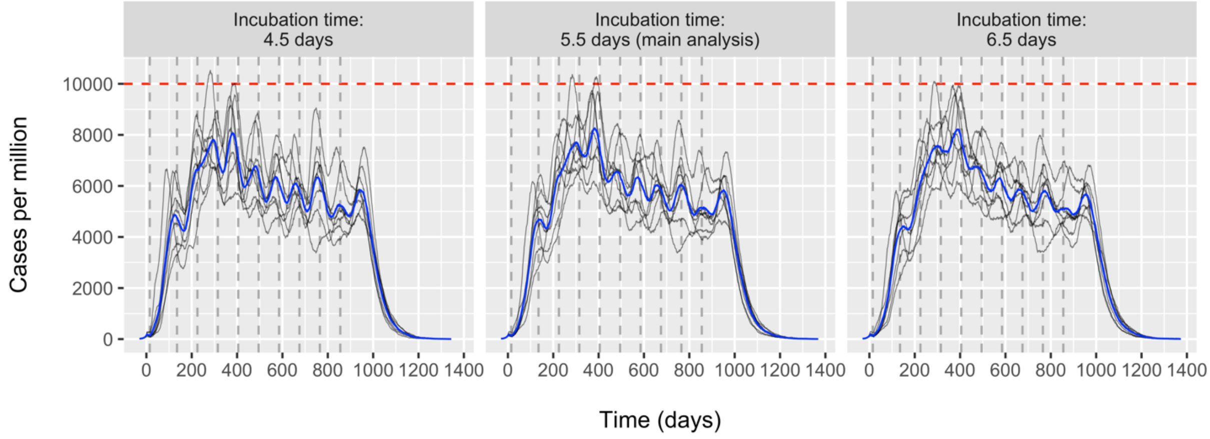

Figure S3.14. Shape of the Weibull distribution for variation in incubation time. Higher values for shape indicate lower variation.

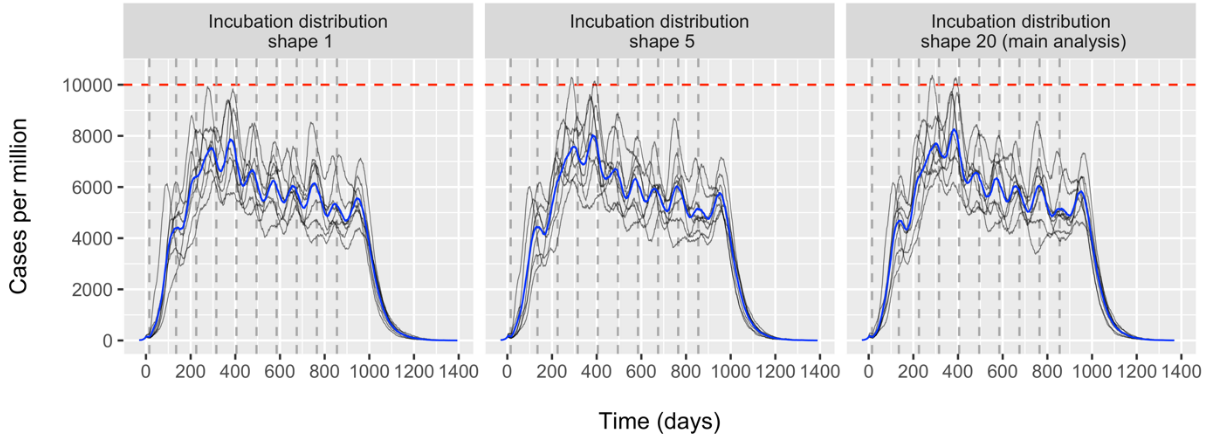

The following two figures cover factors that are not readily controllable: the case incidence when control was originally initiated (a moment that has already passed in most countries), and inter-individual variation in adherence to control measures leading to variation in reductions of individual contact rates. Figure S3.15 clearly indicates that the case incidence that triggered initial nation-wide control measures does not influence the impact of phased lifting of control.

Also, the degree of individual variation in adherence to control measures is of little impact on the effect of phased lifting of control (Figure S3.16), as long as the average reduction in contact rates is sufficient (Figure S3.3). Higher inter-individual variation leads to a flatter, but more erratic epidemic curve.

Figure S3.15. Threshold for cumulative incidence of cases of infection for starting the first control measures (country-wide).

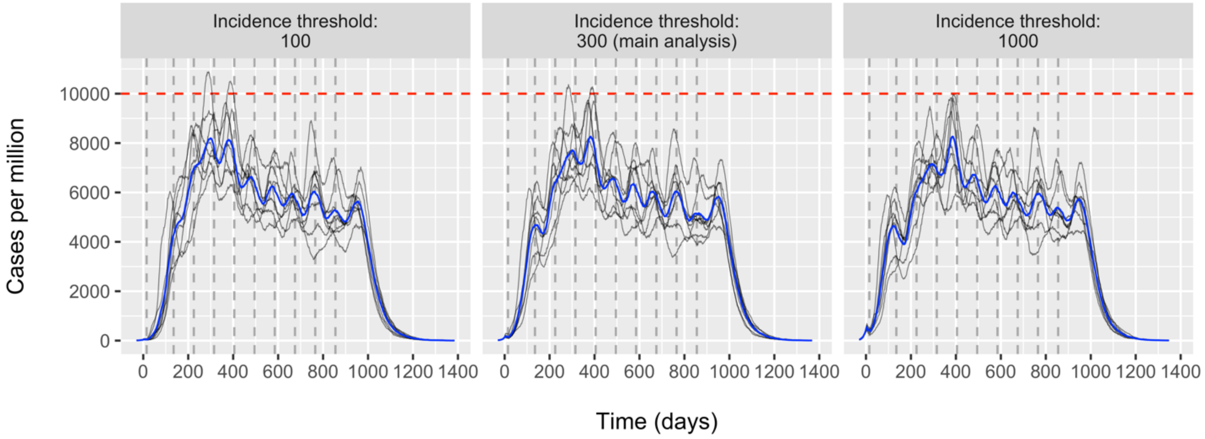

Figure S3.16. Inter-individual variation in the effect of control measures on contribution and exposure to transmission. In all panels, control measures are assumed to reduce transmission to 25% on average. In case of no variation, all individuals in the supercluster (100%) experience a reduction in transmission to 25% of the original level. Low variation means that 95% of the population experiences a reduction in transmission to 50% or lower (the average is still 25%). Moderate variation means that 95% of the population experiences a reduction to 75% or lower. Maximum means that 75% of the population experiences no transmission at all (reduction to 0%) and 25% do not experience any reduction (100%).

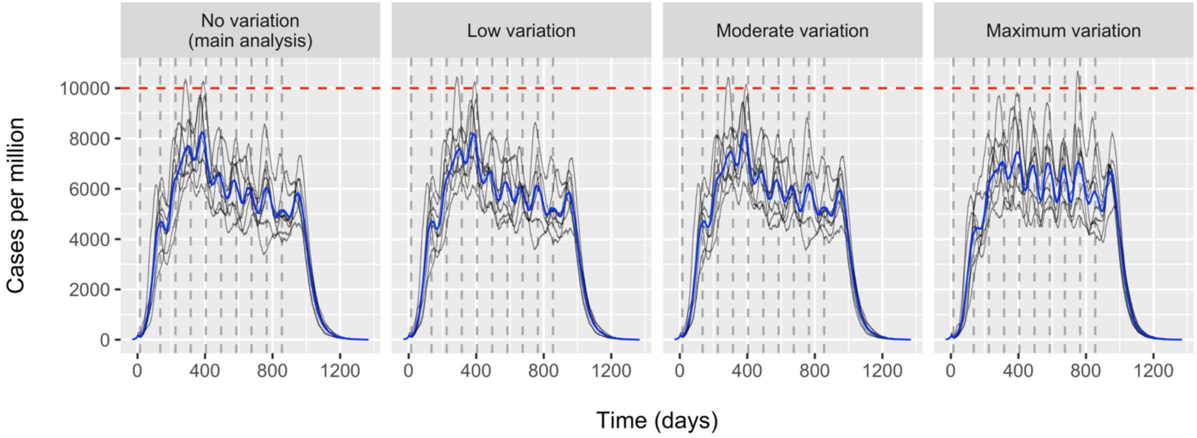

In the rest of the sensitivity analyses, the average contact rate was recalibrated (Table S3.1) such that the predicted initial exponential growth of the epidemic matched that as predicted by a simple homogeneous SEIR model with $R_0 = 2.5$, an average incubation time of 5.5 days, and an average duration of infectiousness of 10 days.

Figure S3.17 shows that a lower level of inter-individual heterogeneity leads to a deviating epidemic pattern compared to moderate and high levels of variation. This can be explained by the fact that for a model with a lower level of heterogeneity, the average contact rate has to be increased (Table S3.1) to achieve the same initial exponential growth of the epidemic (corresponding to $R_0 = 2.5$), thus leading to an overall higher number of cases. Similarly, a lower level of assortative mixing (Figure S3.18) requires that the average contact rate is increased, resulting in more cases overall and a peakier distribution of cases over time. Therefore, the shape of local outbreaks observed during actual implementation of a phased lift of control may inform us about the degree of assortative mixing.

Figure S3.17. Level of inter-individual heterogeneity in relative contact rates. Panel headers indicate the degree of heterogeneity in terms of the relative difference between the 2.5th and the 97.5th percentiles of the distribution of individual relative contact rates.

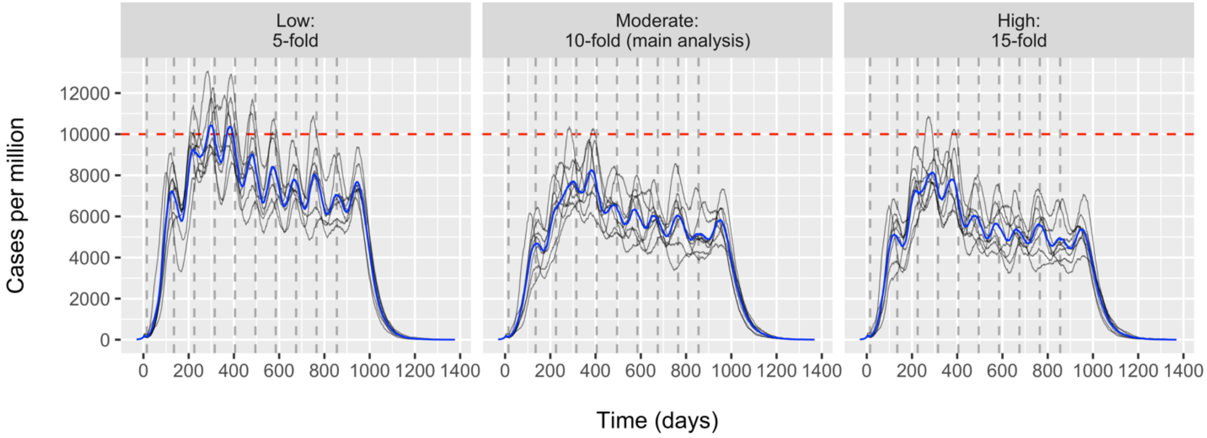

Figure S3.18. Assortative mixing. Panel headers indicate the degree of assortative mixing in terms of the relative difference between the 2.5th and the 97.5th percentiles of the distribution of average relative contact rates per cluster.

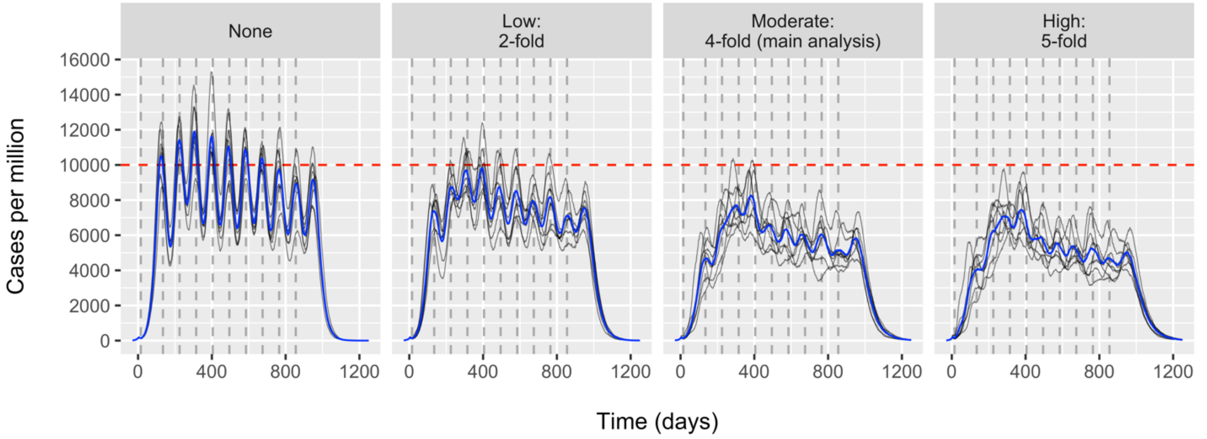

Figure S3.19 illustrates the impact of one of the least well-known factors in COVID-19 transmission: geographical mixing. Clearly, a higher level of mixing within superclusters and within the country as a whole increases the initial speed of the epidemic, leading to a lower calibrated value of the average contact rate (Table S3.1) and fewer cases overall (left panel). Conversely, if transmission mainly occurs within clusters, the average contact rate had to be calibrated to a higher value to ensure that the infection can spread across the supercluster and the population as a whole. This leads to more cases during the entire epidemic and a higher risk of exceeding the health care system's capacity. This risk should be mitigated by employing an adaptive strategy, in particular by increasing the interval between lifting control in successive superclusters (Figure S3.5) and/or by defining more superclusters of smaller size (Figure S3.2).

Figure S3.19. Geographical mixing of individuals in different clusters and superclusters. Panel headers indicate the geographical mixing pattern in terms of the relative exposure and contribution of individuals to transmission at the level of cluster, superclusters, and the population as a whole (e.g. 90-5-5).

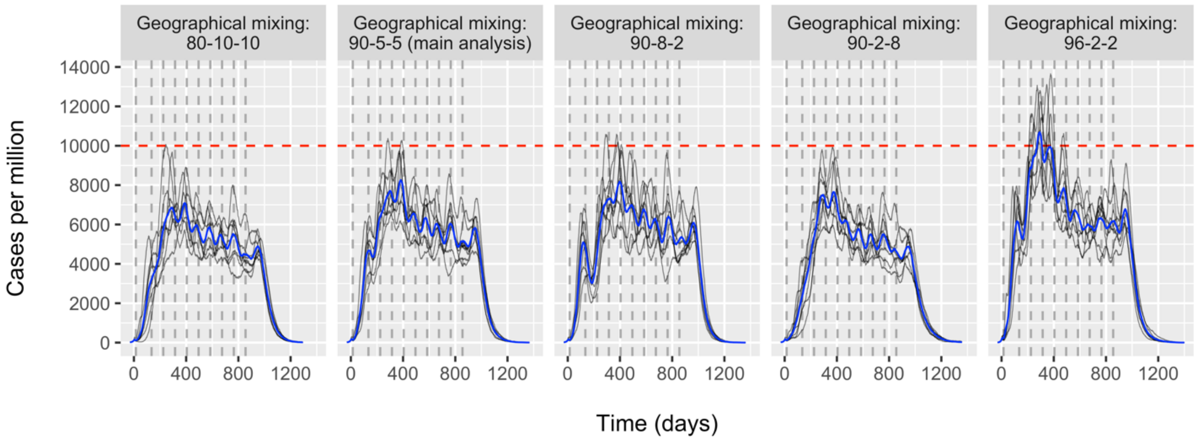

Table S3.1. Overview of recalibrated average contact rates β used in sensitivity analyses and corresponding proportion of immune individuals and the average relative contact rate of susceptible people remaining at the end of a phased lift of control strategy.

Scenario	Contact rate β	Proportion immune (%)	Average relative contact rate of susceptibles
Main analysis			
Reproduction number $R_0 = 2.5$, moderate inter-individual heterogeneity ($\alpha = 3.4$, i.e. 10-fold difference in the 95%-CI bounds of individual relative contact rates), moderate assortative mixing ($\vartheta = 0.45$, i.e. 3.8-fold difference in 95%-CI bounds of average cluster-level relative contact rates), and geographical mixing ($\theta_{\mathrm{SC}} = 0.05$, $\theta = 0.05$), mean incubation time $\nu_E = 5.5$ days, and mean duration of infectiousness of $\nu_I = 10$ days.	0.1717	56.0	0.70
Sensitivity analyses for variation in individual contact rates			
Low ($\alpha = 6.4$, i.e. 5-fold difference in the 95%-CI bounds)	0.2259	72.3	0.74
High ($\alpha = 2.5$, i.e. 15-fold difference in the 95%-CI bounds)	0.1727	54.1	0.65
Sensitivity analyses for assortative mixing			
None ($\vartheta = 0$)	0.2369	77.8	0.64
Low ($\vartheta = 0.26$, i.e. 2-fold difference in 95%-CI bounds)	0.2081	70.0	0.68
High ($\vartheta = 0.55$, i.e. 5-fold difference in 95%-CI bounds)	0.1617	51.0	0.69
Sensitivity analyses for geographical mixing			
$\theta_{\mathrm{SC}} = 0.10$, $\theta = 0.10$	0.1475	49.4	0.74
$\theta_{\mathrm{SC}} = 0.08$, $\theta = 0.02$	0.1688	56.8	0.70
$\theta_{\mathrm{SC}} = 0.02$, $\theta = 0.08$	0.1559	50.0	0.73
$\theta_{\mathrm{SC}} = 0.02$, $\theta = 0.02$	0.2318	67.2	0.64
