# Supplementary material for: Achieving herd immunity against COVID-19 at the country level by the exit strategy of a phased lift of control
Source: Sci Rep. 2021 Feb 24;11:4445. doi: 10.1038/s41598-021-83492-7 (PMC7904921; doi:10.1038/s41598-021-83492-7)
Supplement: Supplementary file 10 — Supplementary Legends. [file 41598_2021_83492_MOESM10_ESM.docx]

**Achieving herd immunity against COVID-19 at the country level by the exit strategy of a phased lift of control**

Sake J. de Vlas, Luc E. Coffeng*

Department of Public Health, Erasmus MC, University Medical Center Rotterdam, Netherlands

*Corresponding author: [l.coffeng@erasmusmc.nl](mailto:l.coffeng@erasmusmc.nl)

**Supplement 10 – Overview of Supplementary Materials**

Supplementary Information 1: Technical model description (pdf)

Supplementary Information 2: Alternative strategies (pdf)

Supplementary Information 3: Strategy adjustments and sensitivity analyses (pdf)

Supplementary Video 1: Animation of the natural course of the epidemic in terms of transmission between clusters and superclusters (.mp4)

Supplementary Video 2: Animation of the natural course of the epidemic in terms of variation in levels of immunity between clusters (.mp4)

Supplementary Video 3: Animation of a phased lift of control in terms of transmission between clusters and superclusters (.mp4)

Supplementary Video 4: Animation of a phased lift of control in terms of variation in levels of immunity between clusters (.mp4)

Supplementary Video 5: Animation of a fast and optimistic phased lift of control in terms of transmission between clusters and superclusters (.mp4)

Supplementary Video 6: Animation of a fast and optimistic phased lift of control in terms of variation in levels of immunity between clusters (.mp4)

Supplementary Legends: Overview of Supplementary Materials (this .pdf file)
